# Cleavage Events and Sperm Dynamics in Chick Intrauterine Embryos 

**DOI:** 10.1371/journal.pone.0080631

**Published:** 2013-11-07

**Authors:** Hyung Chul Lee, Hee Jung Choi, Tae Sub Park, Sang In Lee, Young Min Kim, Deivendran Rengaraj, Hiroki Nagai, Guojun Sheng, Jeong Mook Lim, Jae Yong Han

**Affiliations:** 1 WCU Biomodulation Major, Department of Agricultural Biotechnology and Research Institute for Agriculture and Life Sciences, Seoul National University, Seoul, Korea; 2 Laboratory for Early Embryogenesis, RIKEN Center for Developmental Biology, Chuo-Ku, Kobe, Hyogo, Japan; INRA, France

## Abstract

This study was undertaken to elucidate detailed event of early embryogenesis in chicken embryos using a noninvasive egg retrieval technique before oviposition. White Leghorn intrauterine eggs were retrieved from 95 cyclic hens aged up to 54-56 weeks and morphogenetic observation was made under both bright field and fluorescent image in a time course manner. Differing from mammals, asymmetric cleavage to yield preblastodermal cells was observed throughout early embryogenesis. The first two divisions occurred synchronously and four polarized preblastodermal cells resulted after cruciform cleavage. Then, asynchronous cleavage continued in a radial manner and overall cell size in the initial cleavage region was smaller than that in the distal area. Numerous sperms were visible, regardless of zygotic nuclei formation. Condensed sperm heads were present mainly in the perivitelline space and cytoplasm, and rarely in the yolk region, while decondensed sperm heads were only visible in the yolk. In conclusion, apparent differences in sperm dynamics and early cleavage events compared with mammalian embryos were detected in chick embryo development, which demonstrated polarized cleavage with penetrating supernumerary sperm into multiple regions.

## Introduction

Avian models have tremendous value as ex vivo-model systems for both basic and clinical purposes, enabling monitoring of cell differentiation, transformation, and organogenesis under specific conditions. Nevertheless, limited work has been conducted due to technical difficulties in egg retrieval before oviposition. Furthermore, avian embryos demonstrate discoidal meroblastic cleavage with a large amount of yolk and a small germinal disc [1,2], which hinders monitoring early embryo development. Indeed, very little information on early development before oviposition has been reported [3-6] in comparison with that available after laying of stage X [7]. In this study, we employed a non-surgical intrauterine egg collection by abdominal rubbing [7], which contributes to overcoming current technical limitation. 

Lots of information on cell-fate determination occurring in early embryogenesis was given in a variety of invertebrate and vertebrate species [8-10]. Differing from mammals, polyspermic penetration was physiologically occurred in avian eggs, but detailed observation has not been reported to date. In this study, we employed a non-invasive egg retrieval technique with comparative classifying of egg shall formation and embryogenesis for monitoring details of sperm penetration and early cleavage events.

## Materials and Methods

### Experimental animals

White Leghorn (WL) hens (54–56 weeks old) were used for the collection of intrauterine eggs. We managed chickens according to our standard operation protocol. Relevant experimental procedures for the study were approved by the Institutional Animal Care and Use Committee, Seoul National University before undertaking experiments (SNU-070823-5). 

### Collection of intrauterine eggs from hens

**Figure 1:**
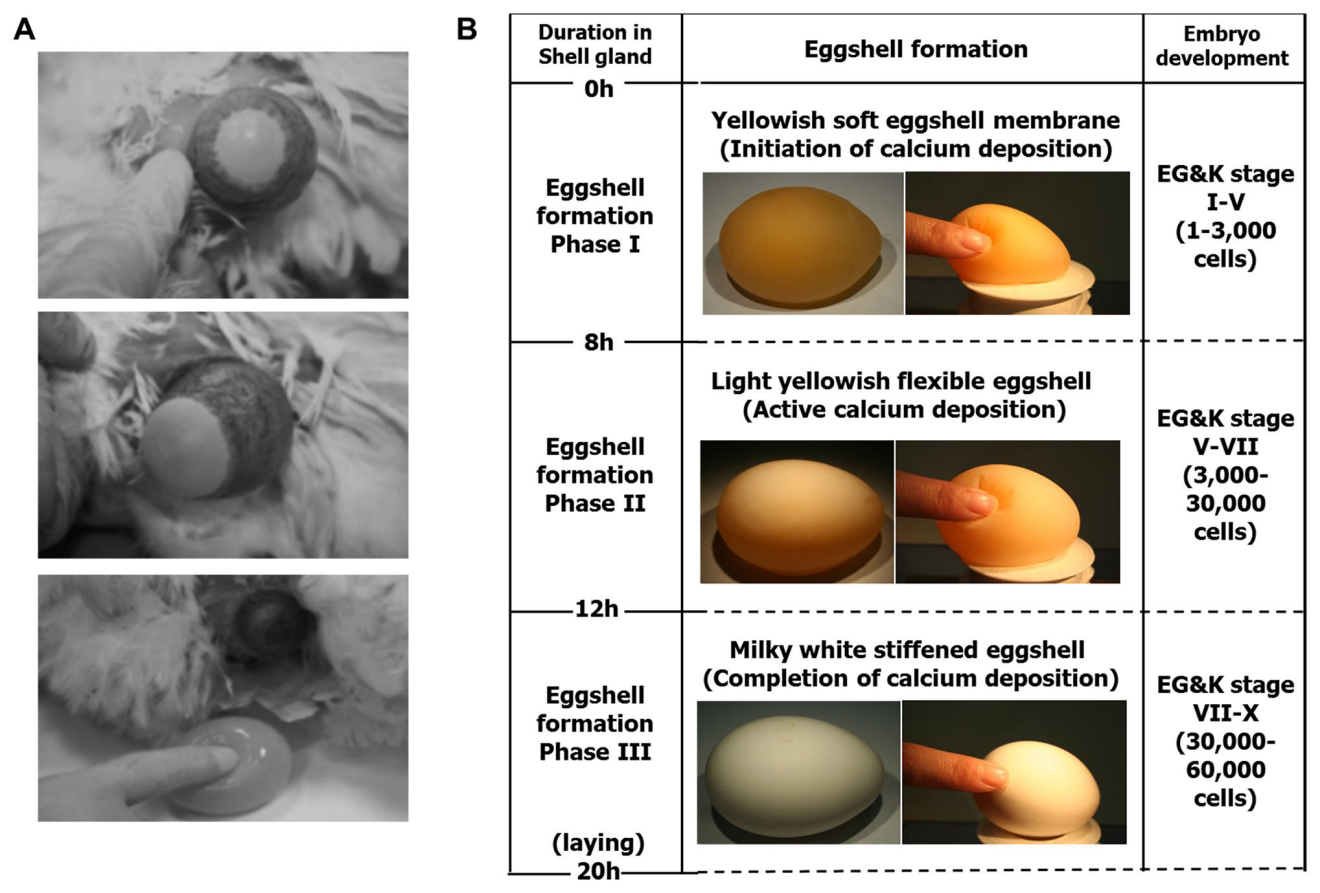
Noninvasive collection and classification of intrauterine eggs by abdominal massage. (A) No surgical manipulation was performed for intrauterine egg retrieval. (B) Phase I, II, and III stages were designated, which were equal to embryonic EG&K stages I–IV, V–VI, and VII–X, respectively. The phase I stage represented as an egg with a yellowish and soft eggshell, phase II was an egg with a light yellow-colored, flexible eggshell, and phase III was an egg with stiffened and calcium-deposited eggshell with a milky-white color.

Intrauterine eggs retrieved from WL hens were harvested by an abdominal massage technique slightly modified from Eyal-Giladi and Kochav [7]. Briefly, the abdomen of hens was pushed gently until exposure of the shell gland, and the surface of the shell gland expanded when an egg was located there for eggshell formation. After expansion of the surface of the shell gland, massage was used to move the egg gently toward the cloaca until the intrauterine egg was released (Figure 1A). 

### Analysis of cleavage stages in the intrauterine embryos

Intrauterine embryos were separated from the egg using sterilized paper [11] and the shell membrane and albumen were detached from the yolk. A piece of square-type filter paper (Whatman, Maidstone, Kent, UK) with the hole at the center was placed over the germinal disc. After cutting around the paper containing the intrauterine embryo, it was gently turned over and transferred to saline buffer to further remove the yolk and the vitelline membrane for embryo collection [12]. Collected embryos were fixed with 4% (w/v) paraformaldehyde in 1× phosphate-buffered saline (PBS) and the fixed embryos were classified according to the cleavage stages proposed by Eyal-Giladi and Kochav [7]. Unfertilized and abnormal embryos were identified by the morphological criteria of cleavage furrows. 

Photographs of the dorsal part of intrauterine embryos were taken with a stereoscopic zoom microscope (SMZ1000; Nikon Corporation, Tokyo, Japan) and EG&K stage I-II embryo was cultured in Chamlide incubator system (Live Cell Instrument, Seoul, Korea) at 41.5 °C with 5% of CO_2_ gas for live cell imaging. Shell membrane- and albumen-detached eggs were put into the 25 ml plastic cup (40025; SPL Life Sciences, Pocheon, Korea) with 10 ml of albumen on the bottom and the surface area on the top was covered with 3 ml of albumen. For retaining embryo viability, all procedures were undertaken less than five minutes in the heated room (more than 30 °C). Time-lapse images were taken by multi-purpose zoom confocal microscope (AZ100; Nikon Corporation, Tokyo, Japan).

### Phalloidin and DAPI staining of intrauterine embryos

After fixation with 4% paraformaldehyde, the intrauterine embryos were washed in PBS three times and incubated in 0.1% (v/v) Triton X-100 in PBS (PBST). The fixed embryos were incubated with Alexa Fluor 488 phalloidin (A12379; Invitrogen, Carlsbad, CA, USA) diluted 1:40 in PBST overnight at room temperature. After overnight incubation, the embryos were washed three times in PBS and mounted with Prolong Gold antifade reagent with 4',6-diamidino-2-phenylindole (DAPI) (P36931; Invitrogen). The stained embryos were observed under a fluorescence microscope (Ti-U; Nikon Corporation). In addition, the intrauterine embryos were embedded with paraffin and sectioned (12 µm) using a microtome and after being mounted with Prolong Gold antifade reagent with DAPI, the embryonic nuclei were evaluated under a fluorescence microscope.

### Statistical analysis

Statistical analyses were performed using the Student t test in SAS version 9.3 software (SAS Institute, Cary, NC). The significance levels between control and treatment groups were analyzed using the general linear model (PROC-GLM) in SAS software. Differences between treatments were deemed to be significant when P was less than 0.05.

## Results

### Retrieval of intrauterine eggs

The general procedure for the noninvasive collection of intrauterine eggs by abdominal massage is shown in Figure 1. This procedure resulted in minimal stress to the hens, which continued to lay eggs from the second day after harvest. Ninety-five WL hens at 54-56-week-old were provided for egg retrieval, and intrauterine eggs were retrieved from all hens. Among the 95 collected embryos, 38 were of EG&K stage I, 26 of stage II, 11 of stage III, 13 of stage IV, and 7 of stage V. In total, 67.4% of the harvested intrauterine embryos were classified as early EG&K stages I-II. Intrauterine eggs can be divided into three categories based on morphological characteristics (Figure 1B): eggs with a yellowish soft eggshell membrane of EG&K stages I-V, eggs with a light yellowish flexible eggshell of EG&K stages V-VII, and eggs with a milky-white stiffened eggshell of EG&K stages VII–X. Eggshell formation advanced gradually in the shell gland. The calcium-deposited eggshell was well formed during EG&K stages V-VI (8 h in the shell gland), hardening of the eggshell was observed at EG&K stage VII, and eggshell formation was complete by EG&K stages IX-X. Overall times to retrieve each stage were expected to be 0-8, 8-12, and 12-20 h after entering into shell gland for phases I, II and III, respectively.

### Morphogenesis of cleavage furrows in intrauterine embryos

**Figure 2:**
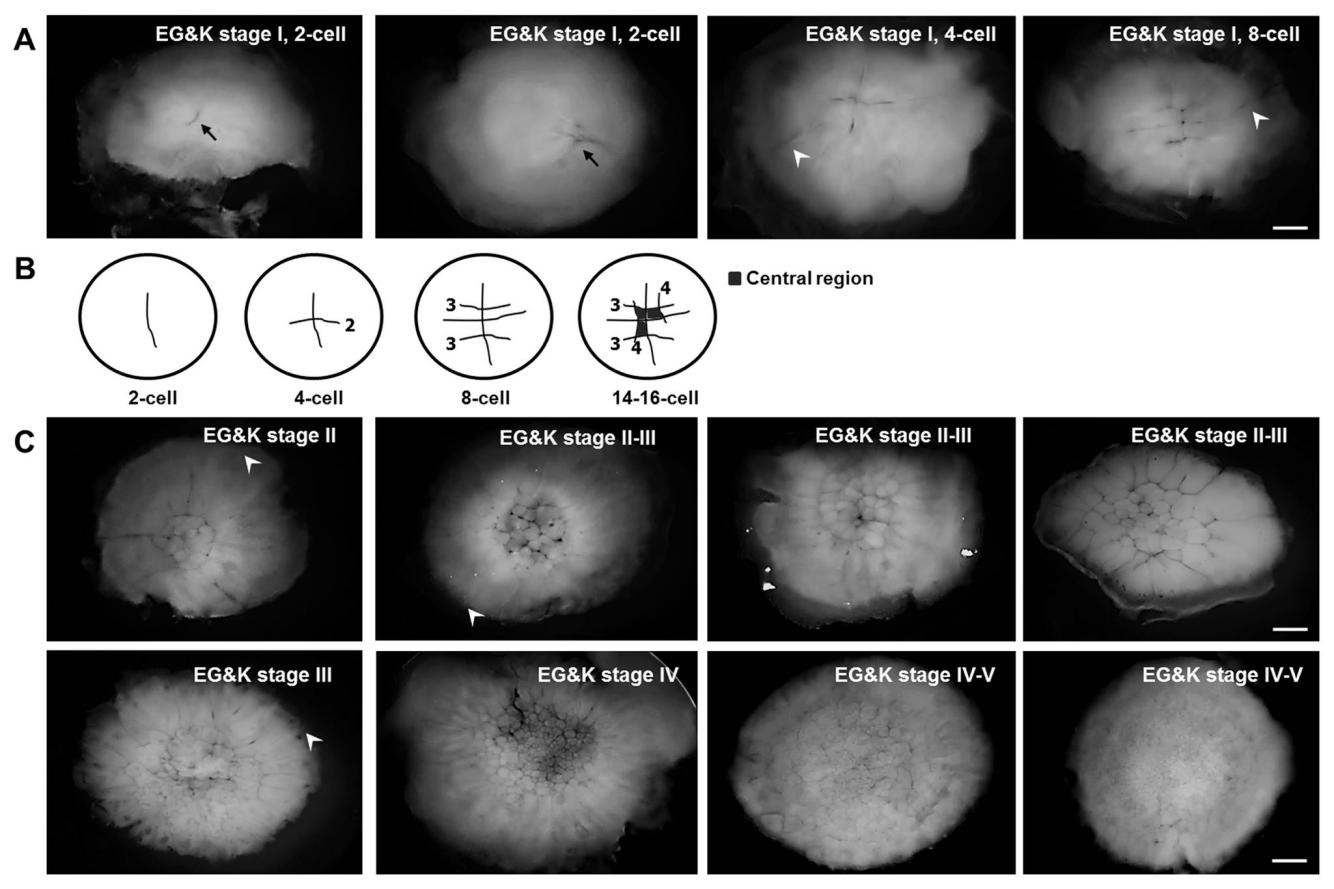
Cleavage of harvested phase I stage eggs in vitro. (A) Formation of cleavage furrows in the EG&K stage I, 2–8-cell embryos. Asymmetric divisions with synchronized cleavage at the early EG&K stage I were observed. (B) Schematic diagram showing the pattern of early cleavage in 2–8-cell embryos. The first two divisions were synchronized and the initial cruciform cleavage yielded four nonpolar preblastodermal cells. (C) Cleavage of EG&K stage II–V embryos. Cleavage proceeded in a radial manner from the cleavage initiation region. Black arrows indicate the first cleavage furrow, and white arrowheads denote cleavage furrows in the peripheral area (scale bar = 500 μm).

**Figure 3:**
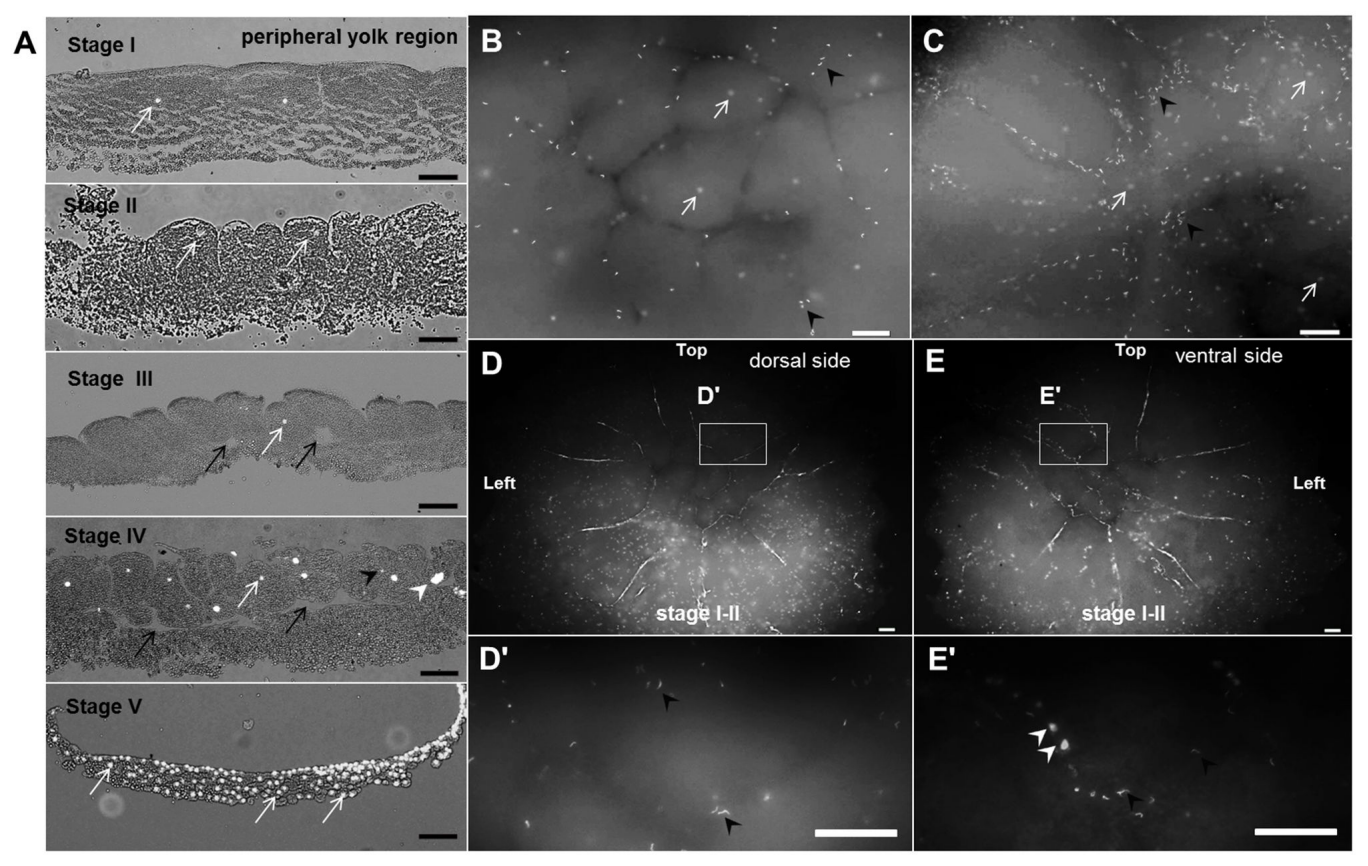
Spatial distribution of condensed and decondensed sperm heads. (A) Relative position of embryonic and sperm nuclei during development (sectioned view). Condensed sperm heads were visible on the dorsal side of EG&K stage I (B) and III (C) embryos. (D) The majority of condensed sperm heads were visible on the dorsal side of EG&K stage II embryos, while decondensed sperm heads were observed on the ventral side (E). In a few embryos, a few condensed sperm heads were also visible on ventral side (E). (D', E') Higher magnification images of (D) and (E). White and black arrows indicate embryonic nuclei and subgerminal cavities, respectively, while white and black arrowheads indicate decondensed and condensed sperm nuclei, respectively. Decondensed sperm heads were present primarily in the yolk and cytoplasmic areas (scale bars = 100 μm).

Of the 38 EG&K stage I embryos collected from the shell glands, five were undergoing the first cleavage (Figure 2A). The first cleavage furrow was observed in the central region, while a few showed the initiation of cleavage in the peripheral area. Six of the 38 underwent synchronous cleavage up to the third cleavage, perpendicular to the previous cleavage furrow. The fourth cleavage separates central and peripheral cells (schematic diagram; Figure 2B). Distinguishable from the main cleavage furrows formed in a cruciform manner, peripheral cleavage furrows were formed at the embryo boundary until EG&K stage V (Figure 2A, C). The peripheral furrows disappeared gradually after EG&K stage V and became invisible. During cell divisions between EG&K stages I and V, cell size decreased gradually and was approximately tenfold smaller (from 250-300 to 15-40 μm) at EG&K stage V than that of the first cleavage stage (Table 1). As shown in Table 1, preblastodermal cells, indicating completely closed cells detached from the yolk, were detected from EG&K stage III, but the size varied due to rapid cleavage after EG&K stage II. The subgerminal cavity was initially formed with completely closed cells beginning at EG&K stage III. At EG&K stage IV, the central cells began to form cell layers, and three to six cell layers were detected at EG&K stage V; at this stage, preblastodermal cells were observed in both the central and the peripheral regions (Figures 2C, 3A).

**Table 1:** Early morphogenesis of chick embryos before oviposition.

	**EG&K stage**				
	**I**	**II**	**III**	**IV**	**V**
Duration in shell gland (h)	0–1	2	3–4	5–7	8–9
Preblastodermal cell size (μm)	250–300	90–200	80–150	60–100	15–40
No. of cell layers	1	1	1	2–3	3–6
*Preblastodermal cell formation	Only laterally closed cells in the center	Only laterally closed cells in the center	Preblastodermal cell formation in central region	Preblastodermal cell formation in central region	Preblastodermal cell formation in both central and peripheral regions
Subgerminal cavity	Non-developed	Non-developed	Initially seen	Progressed	Progressed
No. of condensed sperm heads	High	High	High	High	Very low
No. of decondensed sperm heads	High	High	High	High	Very low/not detected

Preblastodermal cell is referred as the completely closed cell detached from the yolk.

**Figure 4:**
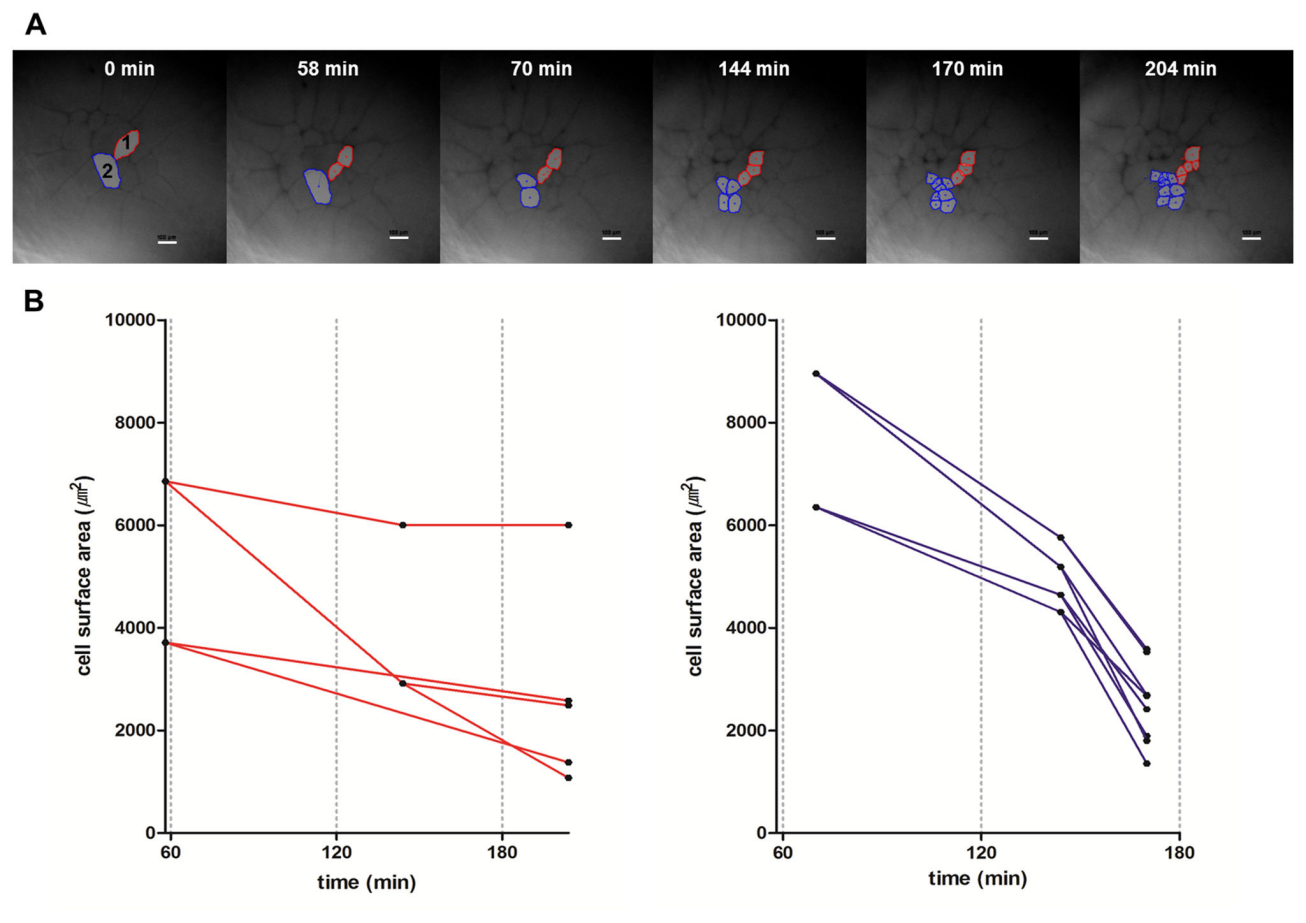
The asynchronous and asymmetric cleavage pattern of the EG&K stage I embryo. The embryo was harvested from the phase I egg stage and cultured in the live-imaging chamber for 4 hours. Time-lapse images were taken by confocal microscope during culture. Cleavage of two adjacent cells at the central region named as ‘1’ (red color) and ‘2’ (blue color) were monitored. (A) Asymmetric division with asynchronous cleavage was notable (scale bar = 100 μm). (B) Changes in cleavage duration and cell surface area in the preblastodermal cells derived from cell number ‘1’ (left) and cell number ‘2’ (right) (X axis = time after culture, Y axis = cell surface area, μm^2^). Data demonstrated both the size of preblastodermal cells and cleavage duration were decreased as the cleavage was progressed.

To further examine cell division, time-lapse live-imaging of the cleaving embryo (EG&K stage I-II) was taken (Figure 4). Cleavage of two laterally closed cells at the central region, which were indicated as ‘1’ and ‘2’ in the first panel of Figure 4A, was monitored during 4 hours of culture. Asymmetric division with asynchronous cleavage was notable in the observation of two cells. The cell surface area of the cell number ‘1’ was 11258.92 μm^2^ at onset of culture and those of its daughter cells were 6855.68 and 3711.55 μm^2^ at 58 minutes after culture, that indicated asymmetric division in each of the two cells (Figure 4B left). In terms of cleavage duration, the second division in one of daughter cells of the cell number ‘1’ completed at 144 minutes after the onset of culture, while that in the other daughter cell completed at 204 minutes after the onset of culture, that indicated asynchronous division (Figure 4B left). The cell number ‘2’ also showed asymmetric division during culture (Figure 4B right).

**Figure 5:**
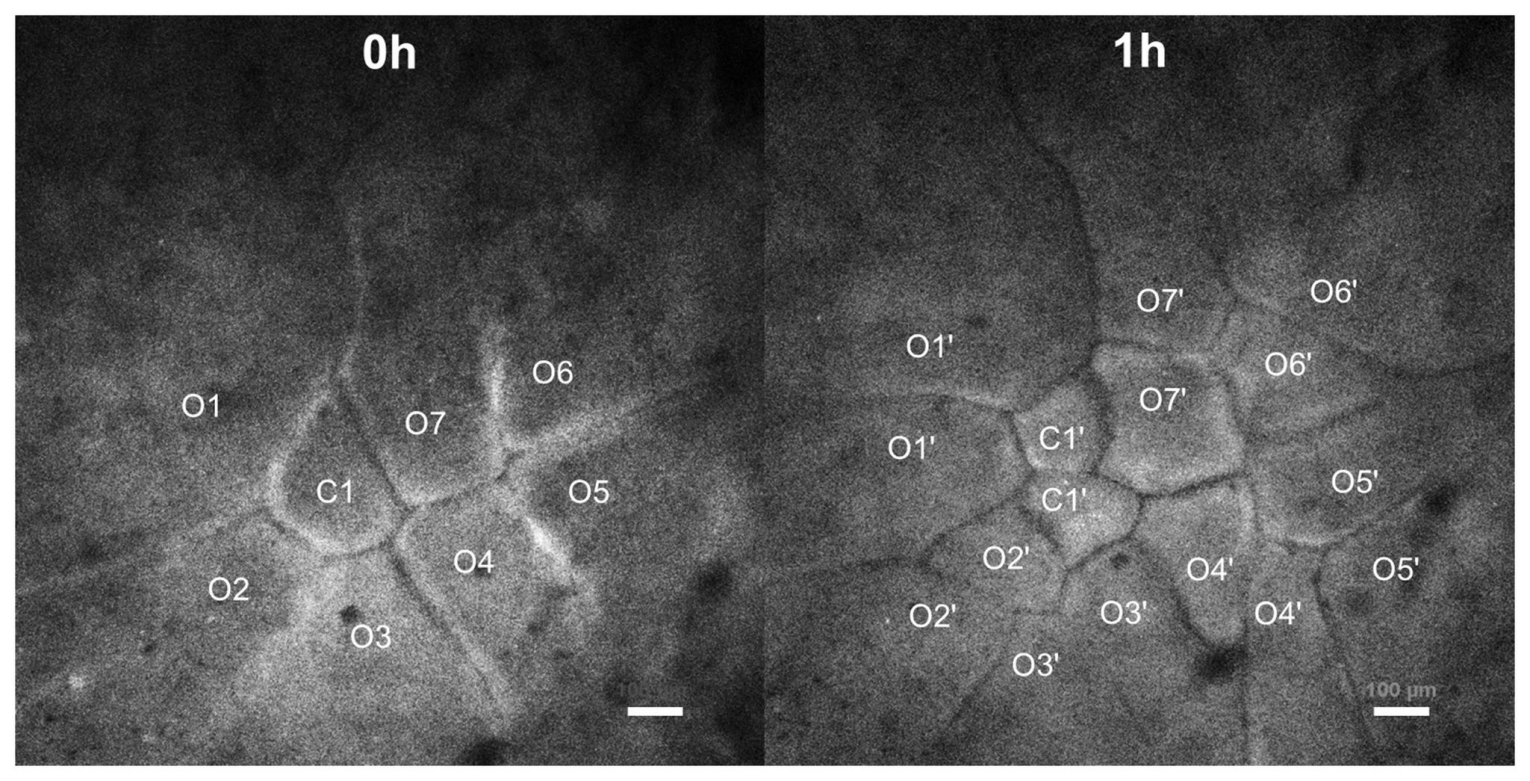
Time-lapse observation on the cleavage of the EG&K stage I embryo in the phase I stage. The embryos were harvested from the phase I egg stage and cultured in the live-imaging chamber. Time-lapse images were taken by confocal microscope during culture. One closed cell (C1) and seven open cells (O1-O7) were present at 0 min and became six closed cells and ten open cells after sixty minutes. The open cells at 0 min divided in two ways; cells labeled O1, 3 and 5 made two open daughter cells, while cells labeled O2, 4, 6 and 7 made one open cell and one closed cell after one cleavage cycle, indicating the division direction of open cells are not fixed (scale bar = 100 μm).

To trace the division direction of open cells, time-lapse live-imaging of the total three cleaving embryos (EG&K stage I) was taken and the one representative embryo is shown in Figure 5. The embryo had total eight cells including one closed cell and seven open cells and the daughter cells were traced during one cleavage cycle. Two kinds of division of open cells were observed. The cells labeled O1, 3 and 5 made two open daughter cells. However, the cells labeled O2, 4, 6 and 7 divided asymmetrically and made one closed cell and the other open cell. The asymmetric division of open cells was observed in all three embryos. The abnormal embryo development and cell apoptosis were not observed during at least 4 hours of ex ovo culture.

### Localization of F-actin to the cleavage furrows and division patterns in intrauterine embryos

**Figure 6:**
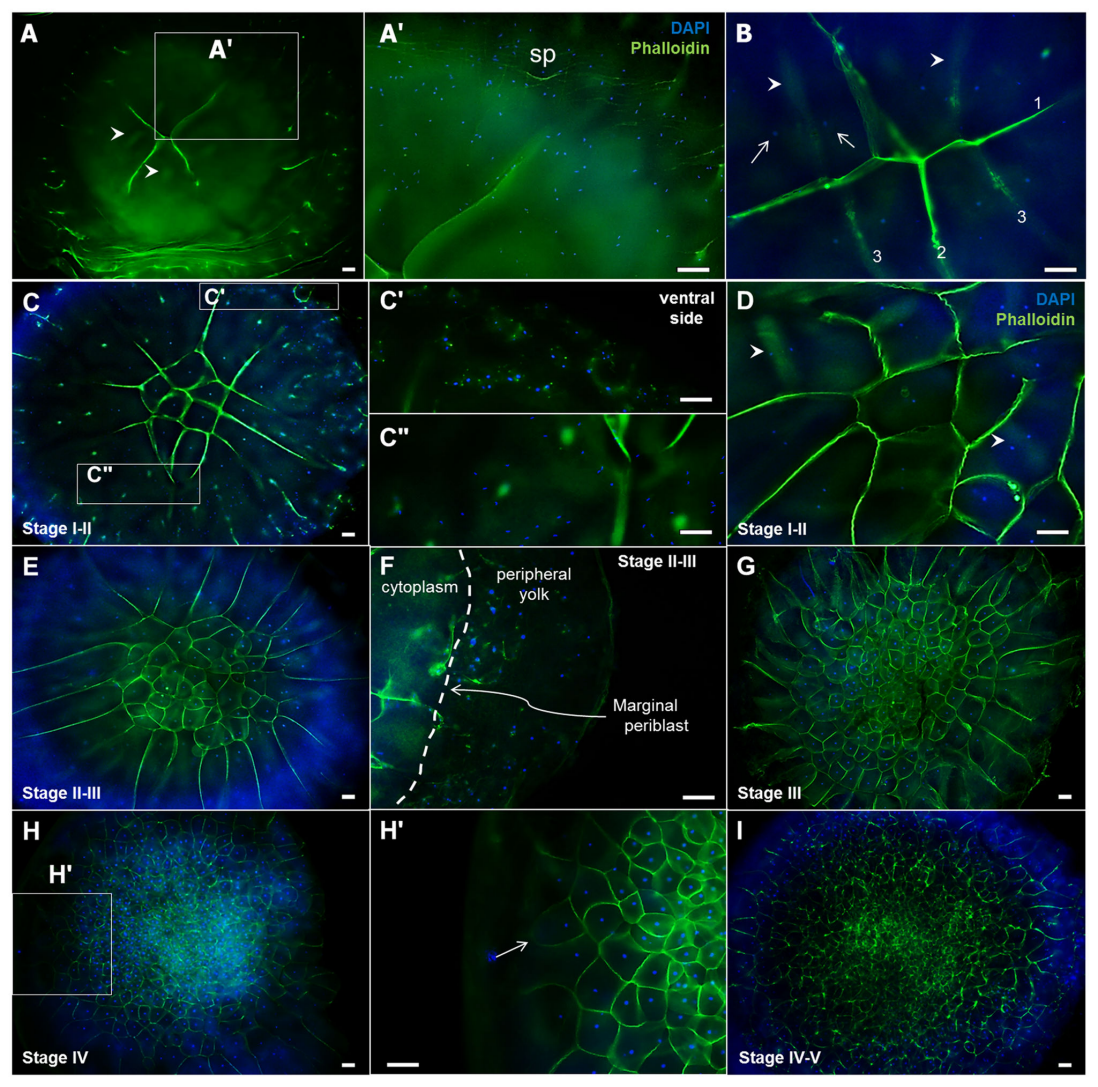
Cleavage pattern in EG&K stage I-V embryos, detected by phalloidin staining. Cleavage of 4-cell embryos was monitored after being harvested (A), and the upper right area (A') was magnified to show many sperm heads appearing as blue spots (Sp: sperm). Cleavage of 8-cell embryos was monitored after being harvested (B). Mitotic nuclei stained with DAPI were observed before cleavage furrow formation (arrows), and the furrow formed after detection of mitotic nuclei (arrowheads). New cleavage furrows developing between two daughter nuclei were observed from the ventral, rather than the dorsal side, showing completion of diakinesis before cytokinesis. The order of cleavage furrow formation was indicated in Arabic numerals (B). (C, D) Cleavage of EG&K stage I-II embryos were monitored. Multinuclear preblastodermal cells having two daughter nuclei were detected, while due to vigorous proliferation, the size of preblastodermal cells in the cleavage initiation region was smaller than that of the cells in the peripheral region at the initial cleavage stages. Decondensed sperm heads were visible on the ventral side of the embryos (C') and condensed sperm heads were visible on the dorsal side (C") and formation of the large number of cleavage furrows before cytokinesis was visible primarily in the peripheral region (C). Formation of cleavage furrows with mitotic nuclei in stage II was visible (arrowheads in D). (E, F) EG&K stage II–III embryos had many decondensed sperm heads, considered to be penetrated sperm, in the peripheral yolk part. (G, H) Image of EG&K stage III and IV embryos and mononuclear preblastodermal cells were visible. Less formation or closing of cleavage furrows was notable in the peripheral region (arrows) at stage IV (H'). (I) Image of EG&K stage IV–V embryos (scale bars = 100 μm).

Nuclear and F actin staining respectively with DAPI and phalloidin was conducted to examine the cleavage pattern of intrauterine embryos. Strong F actin staining was detected in the main cleavage furrow and in the peripheral area of EG&K stage I embryos (Figure 6). Subsequently, F-actin was detected strongly in the second and third cleavage furrows. The newly developed cleavage furrows appeared not to be initiated from the dorsal surface, but rather from deeper cytoplasmic regions underneath the surface (Figure 6A, B). During this early stage, F-actin-stained cleavage furrows from the center did not reach the peripheral area of the embryos (Figure 6). F-actin-stained peripheral cleavage furrows were formed in an irregular (linear, dot-shaped, circular) manner (Figure 6C). From EG&K stage I, the dividing cells in the center became closed first (Figure 6), whereas the peripheral cells were still open before stage IV. Closed cells in the peripheral area were detected primarily in stage IV, and the majority of cells were completely closed in stage V (Figure 6H, I). Double-staining with phalloidin and DAPI clearly showed cell division patterns in the intrauterine embryos in EG&K stages II–V (Figure 6).

### Embryonic and supernumerary sperm nuclei in the intrauterine embryos

**Figure 7:**
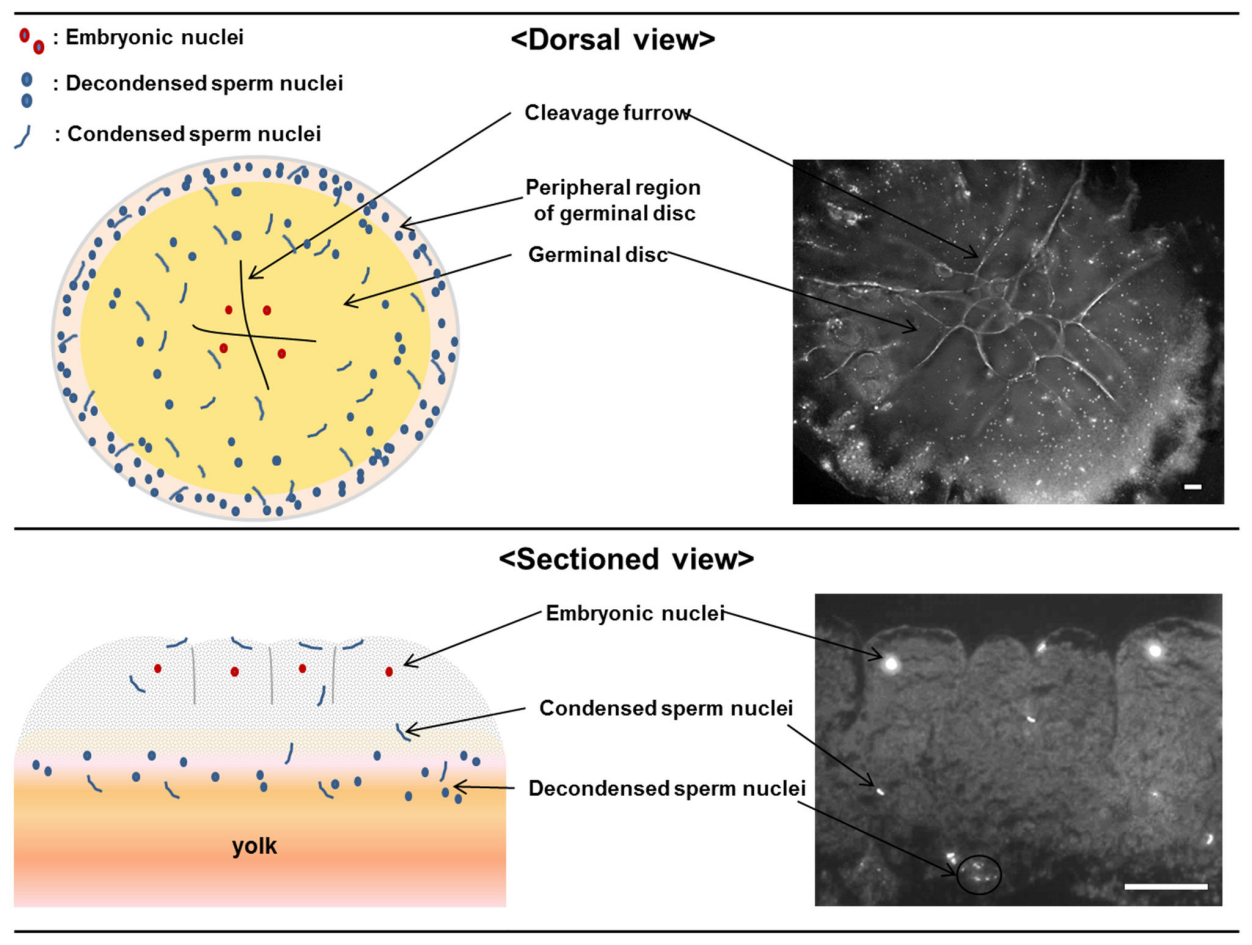
Diagrammatic representation on the position of decondensed and condensed sperm heads. Condensed sperm heads were observed on the dorsal surface in the areas of the germinal disc, cytoplasm, and egg yolk, while decondensed intracytoplasmic sperm heads were observed primarily in the periphery of the egg yolk. Sectioned view (bottom) showed condensed sperm nuclei in the cytoplasm and yolk region. Decondensed sperm nuclei are located in the yolk underneath the cytoplasm (scale bars = 100 μm).

**Figure 8:**
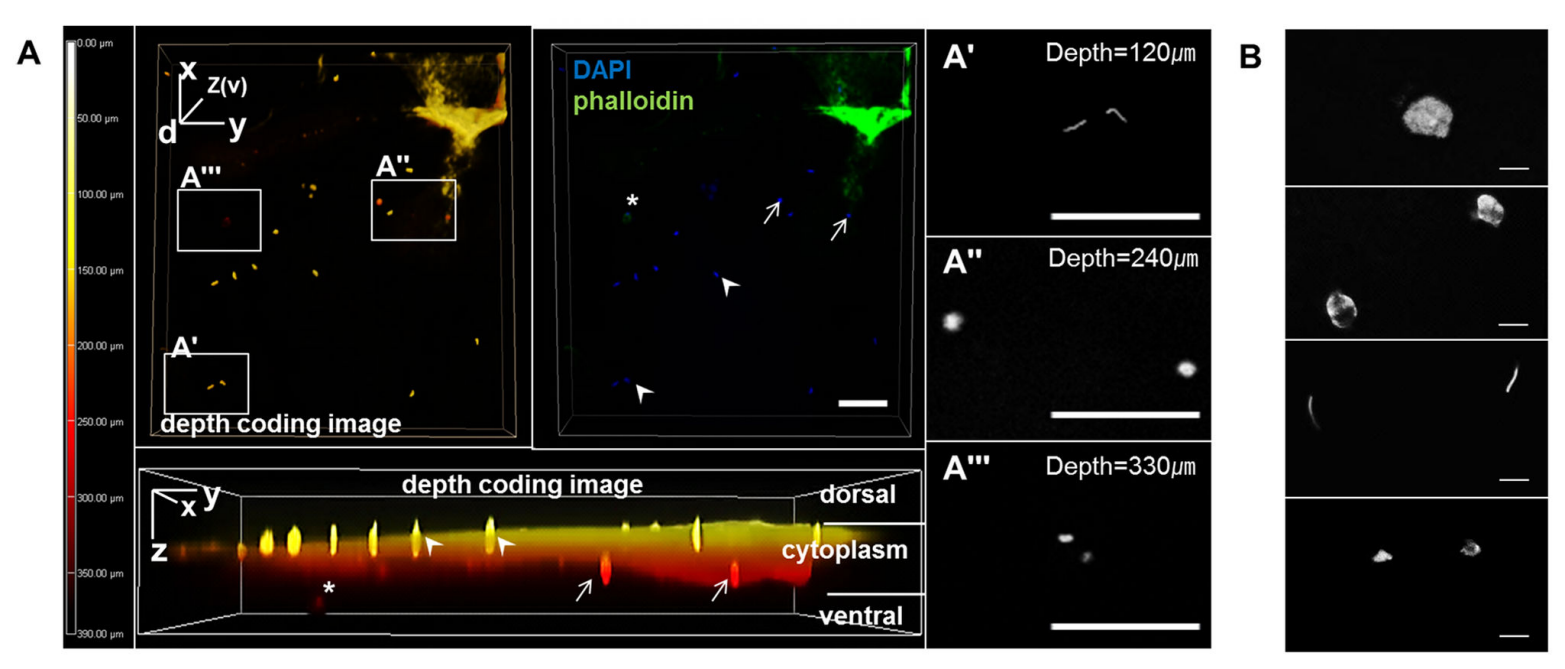
Classification of embryonic nuclei, condensed and decondensed sperm heads by morphology and relative position on the z-axis. (A) Confocal image demonstrated that decondensed sperm heads was present in the yolk under the cytoplasm, while embryonic nuclei were in the cytoplasm. (A'-A'") Higher magnification images of (A) on z-axis of each position. White arrows indicate embryonic nuclei, while arrowheads indicate condensed sperm heads in the surface area. Asterisk denotes decondensed sperm nucleus (scale bars = 100 μm). (B) Morphologies of nuclei present in whole mount embryos stained by DAPI (from top to bottom; embryonic nucleus at interphase, mitotic embryonic nuclei, condensed sperm heads and decondensed sperm heads, scale bars = 10 μm).

Three types of nuclei were observed in the intrauterine embryos according to their morphology, size and position: embryonic (zygotic) nuclei, condensed supernumerary sperm nuclei, and decondensed supernumerary sperm nuclei. Condensed sperm nuclei were mainly present in the dorsal surface and cytoplasm, and rarely in the yolk region underneath the cytoplasm (Figures 3, 7) with a linear shape (Figure 8B), whereas the decondensed sperm nuclei were spread in the peripheral yolk region and yolk region underneath the cytoplasm (Figures 3, 7) with an irregular shape and smaller size compared to embryonic nuclei (Figure 8B). Also, the three-dimensional depth coding image showed that the decondensed sperm nucleus was located under the cytoplasm, while embryonic nuclei were in the cytoplasm (Figure 8A). Less than ten to several thousand condensed and decondensed supernumerary sperm nuclei were detected in the cleavage stages of intrauterine embryos. In particular, the numbers of condensed supernumerary sperm on the dorsal side of EG&K stages I-III embryos ranged from 1 to 10 to more than 1000 per embryo (Table 2). However, late EG&K stage embryos contained very low numbers of supernumerary sperm nuclei. It was obvious that observed sperms were penetrated because the vitelline membrane of all embryos was removed before staining. In the yolk on the ventral side, only decondensed sperm heads were observed in the majority of embryos. In a few embryos, a few condensed sperm heads were also observed on the ventral side as well as decondensed sperm heads. 

**Table 2:** Approximate number of condensed sperm heads on the *dorsal side of EG&K stage I–III embryos after penetration.

**No. of supernumerary sperm**	**1–10**	**10–100**	**100–1000**	**More than 1000**	**Total no.**
No. of embryos	12	22	22	10	66

* The perivitelline membrane was removed from all embryos and only dorsal surface was focused under the microscope for counting DAPI stained nuclei.

**Figure 9:**
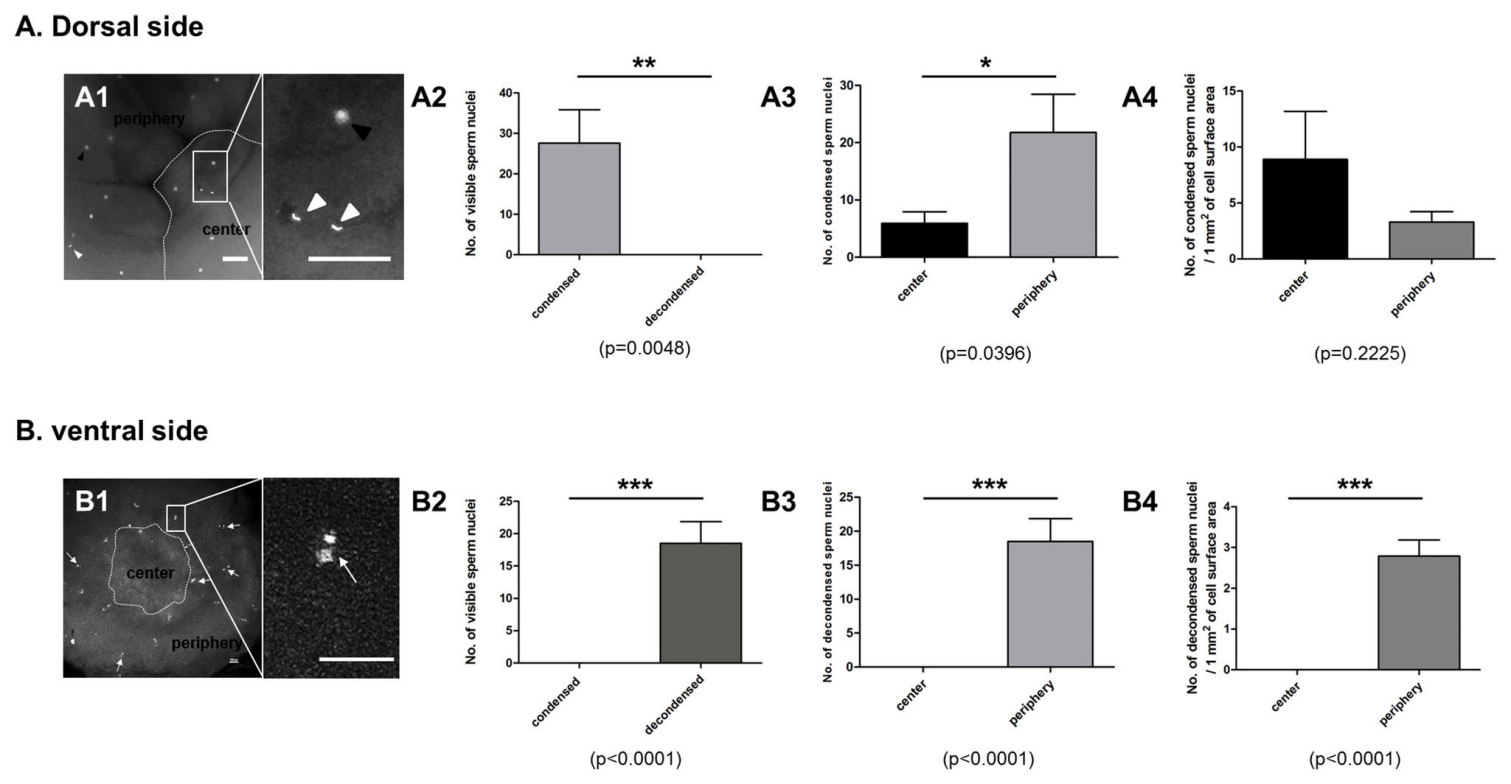
Spatial distribution of supernumerary sperm nuclei on the dorsal side (A) and the ventral side (B) of EG&K stage I-II embryos. (A1 and B1) DAPI stained embryo (scale bars=100μm). On the dorsal side (A1), condensed sperm nuclei and embryonic nuclei were detectable while only decondensed sperm nuclei were detectable on the ventral side (B1). White arrowheads and black arrowheads in A1 indicate condensed sperm nuclei and embryonic nuclei, respectively. Arrows in B1 indicate decondensed sperm nuclei. White dotted line indicates the boundary between the center and the periphery region. The center region and the periphery region were designated for laterally closed cells and open cells, respectively. (A2 and B2) The number of condensed and decondensed sperm nuclei on each side. (A3 and B3) The number of condensed and decondensed sperm nuclei on the center region and the periphery region. (A4 and B4) The number of condensed and decondensed sperm nuclei per 1 mm^2^ of cell surface area. Total eight embryos were used for the experiment (n=8).

To examine the spatial distribution of supernumerary sperm nuclei, condensed and decondensed sperm nuclei on the dorsal and ventral side of EG&K stage I-II embryos were counted respectively (Figure 9). On the dorsal side, condensed sperm nuclei and embryonic nuclei were detectable while decondensed sperm nuclei were present on the ventral side (Figure 9A2, 9B2). Also, the mean number of condensed sperm nuclei was significantly higher on the periphery region than center region (Figure 9A3). The number of condensed and decondensed sperm nuclei per 1 mm^2^ of cell surface area was shown in Figure 9A4 and 9B4. 

## Discussion

The finding of this study clearly demonstrated different aspects of sperm penetration and embryo cleavage between birds (chicken) and mammals. There was a unique, radiating progress of preblastoderm furrowing which showed different furrowing status between the dorsal and the ventral surfaces. Interestingly, different status of spermatozoa penetrated into egg preblastoderm was detected and uneven distribution of condensed and decondensed sperm heads were detected in central (furrowing-completed, cleavage-initiated region) and peripheral (furrowing-incomplete, cleavage-progressing region) parts of the preblastoderm. Although it was not certain whether supernumerary sperm move from the center toward the periphery, it was obvious that they were abundant in the periphery than the center. To clarify the exact function of supernumerary sperm on cleavages, what components of sperm contribute to embryos should be identified in further studies. In the yolk on the ventral side, decondensed sperm nuclei were mainly detected, which might imply either the presence of decondensation factor in the yolk or the entry of sperm into the yolk area only through the preblastodermal region. In any case, this is the unique phenomenon in chick embryos, which is not seen in the mammals.

 In this study, we used a modified noninvasive collection method (abdominal massage) for retrieving intrauterine EG&K stage embryos, which was originally reported by Eyal-Giladi and Kochav [7]. Based on this original technique, we provided the detailed information for the classification, which reflected egg shell formation and a compatible comparison was possible between the newly suggested classification and the “conventional” EG&K classification. There has been no classification reflecting both eggshell formation and embryo development. Combining of EG&K classification with eggshell formation, formation of area pellucida begins from EG&K stage VII, thus this stage was the first lineage differentiation in chicken. Calcium-deposited eggshell was formed from EG&K stage V and eggshell hardening was observed from EG&K stage VII. There seems to be a close correlation between eggshell formation and formation of area pellucida. By employing this modified classification, it will be feasible to identify and to collect embryos at various intrauterine stages.

We found a significant difference in the dynamics of the sperm that had penetrated into oocytes and in early cleavage. Polyspermic fertilization, with large numbers of decondensed or condensed sperm in an oocyte, was observed. Differing from mammals, many unfertilized supernumerary sperm heads were observed in the yolk area, as well as in the cytoplasm. Several sperm heads in the yolk were undergoing decondensation. The sperm tract from the extracellular space into the yolk was unknown, whether it was direct penetration into the yolk or penetration via the cytoplasm. Decondensed sperm may pass through the cytoplasm during the initial stage of egg development. 

Asymmetric cleavage was initiated as early as from the first cleavage, which triggered radiation-oriented progress from central to peripheral part. Central cells in a cleaving embryo seemed to divide very rapidly while peripheral cells, including open cells, divided relatively very slowly. The peripheral furrowing could be readily distinguished from the central one by their length and origin. The peripheral cleavage furrows formed from the peripheral edge of embryo, elongated toward the center, and were more easily visible from the ventral side; however, they were not detectable after EG&K stage IV. This furrowing-type cleavage yielded lots of differences compared with the cleavage of mammalian embryos. In mammals, asymmetric, polarized cleavage signs the initiation of differentiation, while in chick, each part of the preblastoderm being separated was still connected to each other at the ventral side even after initial furrowing. So, it is difficult to simply reflect the knowledge from the mammals and to further justify the signs of initial differentiation. 

Preblastodermal cell divides rapidly. Bellairs et al. [4] stated that the open cells mitotically divide into two daughter cells: one is laterally closed, and the other is open. One daughter nucleus migrates into adjacent yolk, while the other remains *in situ*. This indicates that the possibility of a different division mechanism in open and closed cells. In this study, however, the open cells observed in the peripheral region did not always generate both closed and open daughter cells. They could divide into two open cells as well as both closed and open daughter cells, indicating that the division direction of open and closed cells are not fixed. However, formation of the subgerminal cavity at the center of EG&K stage III embryo [7] may be an inducible factor for dividing central cells vertically to create two or more layers.

Polyspermy or supernumerary sperm are not common in mammals, whereas they are consistently found in avian species [13]. Chick embryos begin normal development after numerous sperm penetrate the oocyte cell membrane, suggesting that supernumerary sperm may be important to ensure karyogamy [14]. Considering the small area of the germinal disc in relation to the entire ovum of the chicken, polyspermy or supernumerary sperm are necessary to ensure fertilization [13]. Previous reports have shown that low sperm penetration reduces the fertilization rate in chickens [15,16]. Co-localization of decondensed supernumerary sperm in peripheral small cleavage furrows suggested that decondensation of sperm nuclei is a prerequisite for the short-lived supernumerary sperm-associated peripheral cleavage furrows. We found that decondensed sperm were located mainly on the ventral side of the embryos, specifically underneath the cytoplasm, whereas condensed sperm were located mainly on the dorsal side. This might indicate different role of intracytoplasmic, decondensation factors in development of chicken embryos, compared with mammalian embryos.
